# New therapeutic target for osteoarthritis: modulating immune-metabolic aberrations and micromilieu remodeling in underlying bone

**DOI:** 10.3389/fphar.2026.1826153

**Published:** 2026-07-27

**Authors:** Ying Gao, Lan Shen, Xiaomei Su, Mengsi Yang, Kunyuan Man, Yan Sun

**Affiliations:** 1 Hospital of Honghe State Affiliated to Kunming Medical University, Kunming, Yunnan, China; 2 School of Pharmaceutical Sciences, Kunming Medical University, Kunming, Yunnan, China

**Keywords:** immune cells, immune metabolism, macrophage polarization, nanotechnology, osteoarthritis, osteogenic cells

## Abstract

Traditionally, osteoarthritis (OA) has been thought of as a degenerative disease mostly caused by mechanical wear and tear. However, recent research suggests that immunometabolic pathways drive the active process of subchondral bone remodeling. According to this study, the immunometabolic axis is a key regulatory network in the subchondral bone microenvironment of osteoarthritis (OA), facilitating metabolic interaction between osteocytes and immune cells like macrophages. Bone homeostasis is destroyed by immune cell infiltration, which also promotes the release of inflammatory mediators and local metabolic reprogramming. These results support the idea that OA is an immunometabolic disease. Intervening in the immunometabolic network of subchondral bone can be accomplished by focusing on immune cell metabolic pathways, particularly those that control the phenotypic transition of M1/M2 macrophages. It is anticipated that this approach will control the immunometabolic dialogue, slow the advancement of osteoarthritis, end the vicious cycle of inflammatory metabolic disorders, and ultimately offer a new approach to treating osteoarthritis.

## 1 Introduction

One common long-term degenerative joint condition is osteoarthritis (OA). OA is predicted to impact 355 million people globally by 2024, and its incidence will continue to climb every year. Osteoarthritis is more common in women, obese individuals, and those with a history of joint damage (Robinson et al., 2016). Degenerative changes in articular cartilage, along with subchondral bone sclerosis, osteophyte formation, subchondral cystic alterations, thickening of the joint capsule, and injury to muscles and ligaments, are the pathological features of osteoarthritis. These changes eventually jeopardize the entire joint structure by accelerating the deterioration of articular cartilage. (Yao et al., 2023).

While osteoarthritis is still characterized by cartilage degradation, subchondral bone has become more important in recent years. The subchondral bone plate and the underlying trabecular bone make up subchondral bone, which together give the cartilage on top mechanical support (Zhang et al., 2023a). The subchondral bone plate connects to the bone trabeculae below, which are abundant in blood vessels, nerves, and other kinds of bone cells, and supports the articular cartilage upward. According to new research, osteoclast activation may change the kinetics of bone remodeling, which is regulated by local oxygen concentration and H-type blood vessels. This could lead to cartilage malfunction (Hu et al., 2021). The bidirectional interaction between subchondral bone and cartilage is now recognized as a key driver of OA progression and associated pain.

The recently emerging subject of immunometabolism is intimately linked to this complex interaction mechanism (Suri and Walsh, 2012; Hu et al., 2021). This fundamental idea is determined by how the immune system and cell metabolism interact, as suggested by Mapuskar et al. (2025), Franceschi et al. (2000). The metabolic reprogramming of immune cells, such as oxidative phosphorylation and anaerobic glycolysis, is the basis of immunometabolism and serves to meet the energy and biosynthetic material requirements of immune cells. Thus, these pathways directly control the inflammatory response in chronic illnesses and also encourage metabolic reprogramming as fundamental upstream regulators.

The purpose of this review is to reveal how immunometabolism functions as an interacting network inside the subchondral bone microenvironment of osteoarthritis. We will focus on the metabolic interactions between osteocytes and immune cells in subchondral bone. Therefore, we suggest that immunometabolism interventions can function as a novel, targeted therapeutic approach by focusing on the metabolic processes of immune cells. Therefore, it is predicted that this approach will modify the immune response and halt the course of osteoarthritis, providing new research insights and future therapy options.

## 2 Actors in the subchondral bone microenvironment of osteoarthritis

### 2.1 Resident and mobilized immune cells

**FIGURE 1:**
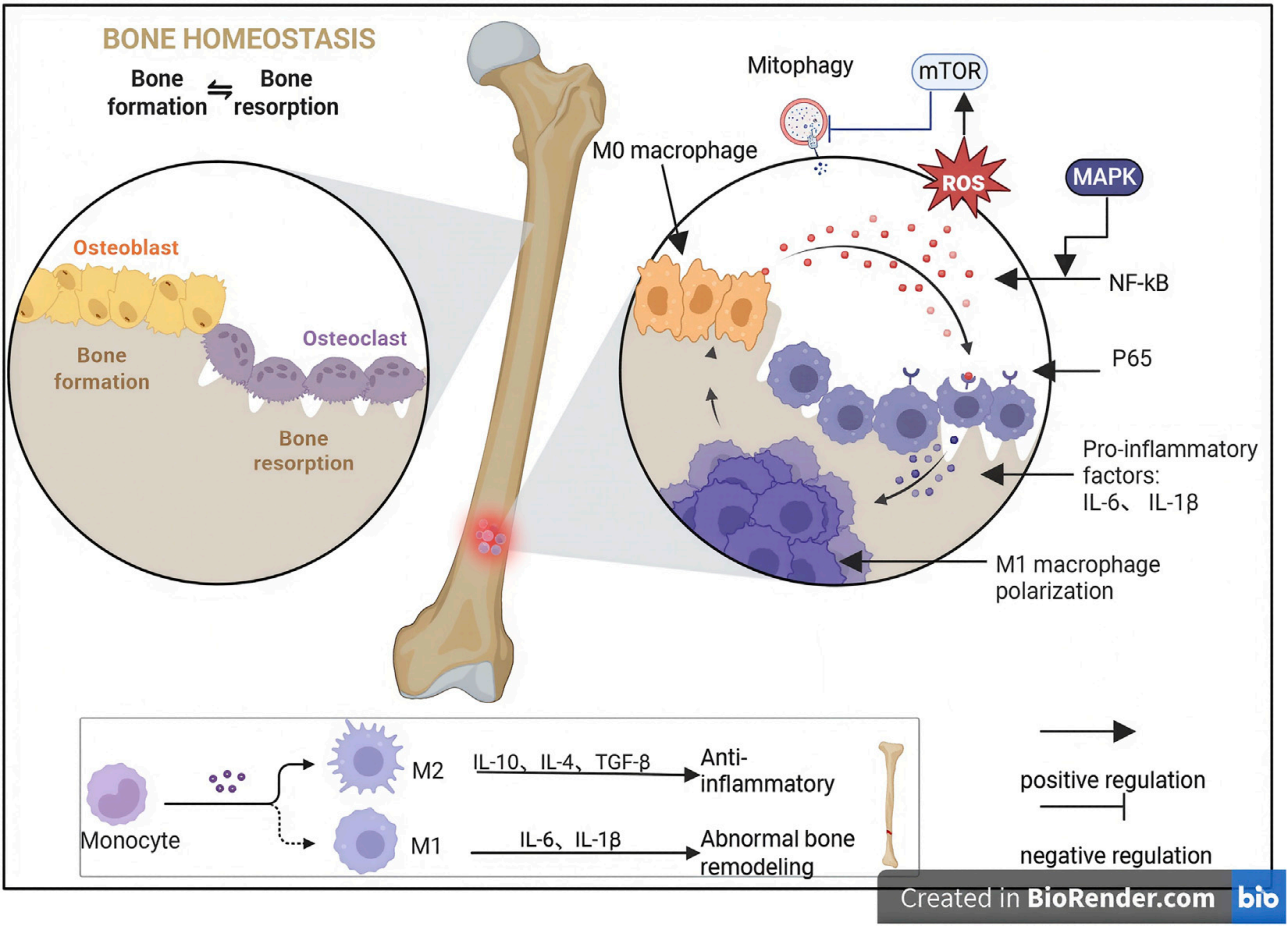
Schematic diagram illustrating the mechanism by which ROS accumulation exacerbates OA pathogenesis through inhibiting mitochondrial autophagy and activating pro-inflammatory pathways. Within osteoarthritis (OA)’s pathogenesis process, reactive oxidative species (ROS) accumulation can activate the mTOR signaling pathway and carry out inhibition of mitochondrial autophagy. At the same time, through synergistic interaction of NF-κB (such as P65 nuclear translocation) and the MAPK pathway, it can drive M1 macrophage polarisation and make cytokines including IL-1β and IL-6 released, hence amplifying the inflammatory response and thus ultimately exacerbating OA development. (OA, Osteoarthritis; ROS, Reactive Oxygen Species; mTOR, Mammalian Target of Rapamycin; NF-κB, Nuclear Factor-Kappa B; MAPK, Mitogen-Activated Protein Kinase; IL-1β, Interleukin-1 BetaIL-6, Interleukin-6).

As important players in the subchondral bone microenvironment of osteoarthritis, macrophages are primarily responsible for immune response regulation and homeostasis maintenance (Table 1). Despite being thought of as the forerunners of the non-specific immune response at first, macrophages can direct and control the overall immune response by secreting a variety of signal molecules (van Kooten et al., 2025; Hsueh et al., 2021; Wu et al., 2020). According to research findings, macrophages primarily contribute to the progression of osteoarthritis through their M1 (pro-inflammatory) and M2 (anti-inflammatory) phenotypes and the inflammatory mediators they produce (Figure 1). By producing pro-inflammatory cytokines including IL-1β and IL-6, M1 macrophages induce subchondral bone inflammation. This imbalance between osteoblasts and osteoclasts results in aberrant subchondral bone remodeling.

**TABLE 1:** Main cells in subchondral bone microenvironment.

Main cells in subchondral bone microenvironment	Type		Function	Metabolic characteristics	References
Immune cells	Macrophage	M1-type macrophages	IL-1β→ inflammation and disturbs bone homeostasis	Oxidative stress and ROS ↑	van Kooten et al. (2025), Wu et al. (2020)
		M2-type macrophages	IL-10↑ TNF-β↑ →tissue repair	Relies on oxidative phosphorylation for energy supply and maintains metabolic homeostasis	van Kooten et al. (2025), Wu et al. (2020)
	T cells		IL-17→ RANKL↑ osteoclast activation	glycolytic activity↑→synthesis of pro-inflammatory cytokines	Yang et al. (2023)
	Neutrophil		Proteases and inflammatory mediators↑→degrade the cartilage matrix	glycolysis↑ →inflammatory mediators	Hsueh et al. (2021)
	Mast cells		histamine↑→pain↓	inflammatory mediators↑→osteoblast metabolism and neural function	Hao et al. (2024)
Osteogenic cells	Osteocyte		Osteocalcin/RANKL→bone remodeling	Mechanical stimulation → metabolic balance between osteoblasts and osteoclasts	Zhang and Wen (2021), El-Masri et al. (2024)
	Osteoblast		Promote bone formation, secrete bone matrix, and maintain bone metabolism	Glycolysis → energy supply; Wnt/β-catenin pathway→ cartilage degradation	Li et al. (2023)
	Osteoclasts		Acidic substances and proteases → subchondral matrix degradation	Mitochondrial oxidative phosphorylation → bone resorption	Lu et al. (2024)
	Mesenchymal stem cells		Differentiate into osteoblasts; secrete IL-10 to inhibit osteoclast activation	Anti-inflammation → oxidative phosphorylation↑; Pro-inflammation → glycolysis↑	Carneiro et al. (2023), Tian et al. (2024)

Conversely, M2 macrophages release anti-inflammatory cytokines such as IL-10, IL-4, and TGF-β to reduce inflammation and promote tissue healing. Therefore, macrophages will experience “over-polarization” toward the M1 phenotype in the OA pathological state, such as reactive oxygen species enrichment. Therefore, a vicious loop of pro-inflammatory activity results from M2 macrophages’ release of anti-inflammatory chemicals being insufficient to stop M1 macrophages’ metabolic inflammatory response (van Kooten et al., 2025; Wu et al., 2020). It is worth noting that M2 macrophages constitute a highly heterogeneous cell population (Table 2). Depending on their induction conditions, key transcriptional regulatory molecules, and secretory profiles, they can be further subdivided into four functionally distinct subtypes: CD206-expressing M2a, CD86-expressing M2b, CD163-expressing M2c, and CD68-expressing M2d (Wang and He, 2022). In particular, the M2a subtype is primarily induced by IL-4 and IL-13, secretes high levels of IL-10 and TGF-β, and is primarily involved in joint tissue repair and the suppression of inflammation (Wang et al., 2019; Zou X. et al., 2025), M2b macrophages (also known as regulatory macrophages) function in opposition to M1 macrophages. Triggered by immune complex signaling, they not only express pro-inflammatory cytokines (IL-1β, IL-6, and TNF-α) but also secrete large amounts of the anti-inflammatory cytokine IL-10, while expressing only low levels of IL-12. Based on their expression profiles of cytokines, chemokines, and other secreted factors, M2b macrophages can modulate immune and inflammatory responses in both breadth and depth. By suppressing immune and inflammatory responses, M2b macrophages promote tumor development and exacerbate infections caused by parasites, bacteria, and fungi. Furthermore, this type of macrophage can mitigate spinal cord injury (SCI) and myocardial ischemia/reperfusion (IR) injury (Wang et al., 2019), M2c macrophages, which are essential for tissue repair, can be induced by IL-10 and glucocorticoids; they secrete CCL-18, IL-10, and transforming growth factor-β, and also possess potent phagocytic capabilities (Wang and He, 2022; Zou X. et al., 2025), M2d cells, also known as tumor-associated macrophages (TAMs), can upregulate vascular endothelial growth factor and inducible nitric oxide synthase, or downregulate tumor necrosis factor-α and arginase-1, playing a critical role in potent immunosuppression, angiogenesis, wound healing, and cancer metastasis (Zou X. et al., 2025).

**TABLE 2:** Overview of Macrophage classification and subtypes.

Cell subtype	Stimulating factor	Distinctive features	Secretory factor	Features	References
M1	—	—	IL-1β; IL-6	An imbalance in the ratio of osteoblasts to osteoclasts	van Kooten et al. (2025), Hsueh et al. (2021)
M2a	IL-4; IL-13	CD206	IL-10; TGF-β	Joint tissue repair and inflammation↓	Wang and He (2022), Wang et al. (2019), Zou et al. (2025a)
M2b	Immune complex signaling	CD86	IL-1β; IL-6; TNF-α; IL-10; IL-12	Tumor progression, parasitic, bacterial, and fungal infections↑; spinal cord injury (SCI) and myocardial ischemia-reperfusion (IR) injury↓	Wang and He (2022), Wang et al. (2019)
M2c	IL-10 Glucocorticoids	CD163	IL-10; CCl-18; Transforming Growth Factor-β	Tissue repair and phagocytosis	Wang and He (2022), Zou et al. (2025a)
M2d	—	CD68	Vascular Endothelial Growth Factor and Inducible Nitric Oxide Synthase↑; Tumor Necrosis Factor-α and Arginase-1↓	Immunosuppression, angiogenesis, wound healing	Wang and He (2022), Zou et al. (2025a)

The metabolic reprogramming process underlying macrophage phenotype has been clarified by recent studies. We have identified the NF-κB and MAPK signaling pathways as the primary mechanisms associated with M1 macrophage polarization (Degboé et al., 2022). The NF-κB signaling pathway is activated in the subchondral bone microenvironment of group OA by endogenous signal molecules associated with damage-related molecular patterns. As a result, this encourages the downstream effector of the NF-κB signaling pathway, its downstream transcription factor p65, to translocate into the nucleus. Simultaneously, it combines with inflammatory mediators such as IL-6 and IL-1β to initiate pro-inflammatory gene expression, initiating the inflammatory response. The signal cascade and NF-κB work together to produce M1 macrophage polarization through the MAPK pathway. By blocking the activity of two signaling pathways, cholesterol sterol, a recently identified regulator of macrophage polarization, can accomplish macrophage metabolic reprogramming. This can lower reactive oxygen species levels, control M1 macrophage pro-inflammatory activity, reduce inflammatory cytokine release, and modify the oxidative phosphorylation process (Wang W. et al., 2025). Additionally, the mTOR kinase pathway is linked to epigenetic modifications in macrophage metabolism. This pathway plays a crucial role in controlling cell metabolism and preventing the onset of autophagy as the central hub that combines internal and external signals. Therefore, the mTOR signaling system modulates autophagy to carry out cartilage repair and control macrophage polarization (Kim and Guan, 2015). ROS-enriched regions may form during the course of OA, which would activate the mTOR signaling pathway, prevent mitochondrial autophagy, and encourage macrophages to adopt the M1 phenotype. On the other hand, autophagy is triggered by blocking the mTOR signaling pathway. In addition to removing damaged organelles and encouraging the NLRP3 inflammasome to degrade, autophagy also prevents the activation and release of interleukin-18 and interleukin-1β, slowing the course of osteoarthritis (Wang W. et al., 2025; Li et al., 2022a).

Important immune cells from several immune system branches, such as neutrophils, eosinophils, and T lymphocytes, are also involved in the development of OA. According to De Lange-Brokaar et al., previous studies have concentrated on the impact of neutrophils on rheumatoid arthritis (RA) (Nedunchezhiyan et al., 2022). Consequently, recent data suggest that neutrophils play a major role in the development of OA. By releasing proteases and inflammatory cytokines, neutrophils actively break down cartilage in the experimental OA mouse model. They also stimulate joint synovial cells to increase the inflammatory response. Additionally, elastase triggers pain signals by activating protease-activated receptor 2 (PAR2) in sensory nerve endings. In the osteoarthritis mouse model, PAR2 gene deletion effectively reduces pain and synovitis (Hsueh et al., 2021).

Mast cells (MCS) play a critical role in the development of structural damage and the exacerbation of pain in OA. Histamine, tryptase, prostaglandins, and leukotrienes are among the inflammatory mediators that are elevated in OA patients due to activated mast cells (Zhao et al., 2022; Kulkarni et al., 2022). The quantity and activity of mucosal cells are clearly higher in osteoarthritis patients than in healthy control individuals, according to clinical test results. As a result, the quantity and the area of synovial angiogenesis, as determined by the Pearson correlation coefficient, have a positive link. Thus, research indicates that mast cells not only directly contribute to the inflammatory response but also impair the integrity of subchondral bone and exacerbate pain by encouraging pathological angiogenesis and innervation (Farinelli et al., 2022; Hao et al., 2024).

T cells, the essential cells of adaptive immunity, especially Th17 cell subsets, are fundamental to the persistent inflammation of rheumatoid arthritis (Li et al., 2017). The specific cytokine IL-17 of Th17 cells can carry out stimulation on synovial fibroblasts and osteoblasts to make them secrete receptor activator of nuclear factor kappa B ligand (RANKL). Under the stimulation of RANKL, osteoclasts accelerate inflammation and promote subchondral bone erosion (Yang et al., 2023).

In sum, the immune cells inside the subchondral bone microenvironment construct a complicated interactive network. Its functional condition is strongly influenced by the local metabolic environment and, conversely, molds the inflammatory process *via* metabolic reprogramming. Therefore, precise immunemetabolic intervention that targets specific pathways in immune cells such as glycolysis and oxidative phosphorylation or their key signaling molecules such as mTOR and NF-κB has risen as one of the most hopeful new directions in osteoarthritis treatment.

### 2.2 Bone lineage cells

Bone cells include bone-building cells, bone-breaking cells, and mesenchymal stem cells. Among these 3 cell types, osteoblasts and osteoclasts are the key mechanosensitive cells of the skeletal system; therefore, they play a very pivotal role in keeping skeletal stability by receiving mechanoreceptive stimuli and making responses to them. According to Wolff’s Law, osteoblasts act as tiny microscopic executors of skeletal mechanical stress, thus promoting the adaptation of skeletal mechanoreceptors to mechanical loads at the cellular level. However, osteoblasts cannot directly feel external mechanical stimuli. Instead, *via* integrin-mediated interaction with connexon 43 hemichannels, they attach themselves to the surrounding cartilage matrix, thereby responding to fluid stimuli and increasing fluid shear stress (Wang et al., 2020). PIEZO proteins are core sensors that change mechanical signals into biochemical signals. Research has discovered that conditional knockout of the Piezo1 gene in mice leads to higher osteoclast activity, stronger bone resorption, and a raised risk of spontaneous fractures under weight-bearing conditions. Hence, the aforementioned studies indicate that, as the core mediator of mechanical loading sensing, the PIEZO protein plays a pivotal role in coordinating crosstalk between osteoblasts and osteoclasts inside the skeleton (Wang et al., 2020; Wang L. et al., 2022). Additionally, osteoblasts and the subchondral matrix form the osteochondral unit. New research shows that MMP3 and MMP13, which have high expression in the chondrocyte OA phenotype, can downregulate the expression of COL2A1, SOX9, and ACAN genes. This means that the chondrocyte phenotype is affected by the osteoblast response. At the same time, under hypoxia condition, β-Catenin in osteoblasts was obviously upregulated, thereby promoting the activation of the Wnt/β-catenin signaling pathway. This will strengthen cell metabolism, speed up the degradation of the extracellular matrix, and at last accelerate the damage to the subchondral bone (Li et al., 2023).

Osteoblasts, serving as mechanoreceptors and core endocrine cells inside healthy bones, can sensitively detect mechanical load changes and secrete signal substances like sclerostin and RANKL to adjust bone remodeling. Sclerostin is a key antagonist for the Wnt signal transduction pathway. Therefore, this material stops Wnt signaling pathway activation *via* combining with LRP5/6 receptors, thus blocking β-Catenin’s accumulation and nuclear translocation, and finally inhibiting osteoblast differentiation and bone formation (Zhang and Wen, 2021). Conversely, the RANKL signaling pathway regulates both aberrant bone resorption and bone rebuilding. According to recent studies, the degree of osteoclast differentiation is determined by the ratio of nuclear factor kappa B activated receptor ligand (RANKL) to decoy receptor osteoprotegerin (OPG). Recombinant OPG (OPG: FC) therapy can accumulate in osteoclast formation sites, but it can also activate osteoclasts and result in a rebound in bone resorption when treatment is stopped. This suggests that the primary mechanism of bone surface reverse elastic absorption is increased osteoclast activation brought on by RANKL expression upregulation and OPG expression downregulation close to the bone surface (El-Masri et al., 2024).

Bone synthesis and resorption maintain a balanced condition in healthy bones, and osteoclast activation is crucial for controlling bone resorption (Lu et al., 2024; Omi and Mishina, 2022). Mature osteoclasts break down the subchondral matrix and accelerate the absorption of bone tissue by secreting acidic substances and proteolytic enzymes. Recent research indicates that when low-density lipoprotein receptor-associated protein 1 (LRP1) is not cleaved by a protease, protease-regulated galectin-3 (Gal-3) can perform a specific interaction with it. One important signaling mechanism for triggering osteoclast activity is the generated Gal-3/LRP1 axis. While inhibiting this route can prevent osteoclast development, activating it might increase bone resorption. This signal transduction pathway’s opening or closing is crucial for controlling osteoclast differentiation, which alters their capacity to resorb bone and ultimately affects bone homeostasis (Zhu et al., 2023).

In addition to having the ability to differentiate in multiple directions, mesenchymal stem cells (MSCs) also exhibit potent immune modulation and anti-inflammatory properties that enable them to control cells in the subchondral environment (Carneiro et al., 2023; Zhang et al., 2024). By interacting with immune cells and secreting bioactive substances, mesenchymal stem cells can reduce an overreaction. For example, macrophages can polarize from a pro-inflammatory M1 phenotype to an anti-inflammatory M2 phenotype in response to IL-10 released by mesenchymal stem cells (MSCs), which lowers the inflammatory environment (Tian et al., 2024). Additionally, the number of neutrophils significantly decreases in a medium rich in mesenchymal stem cells, delaying the course of osteoarthritis (Zhang et al., 2024). According to recent research, synovial mesenchymal stem cells (MSCs) can both migrate to synovial tissue and retain mesenchymal stem cell properties after being implanted into rat osteoarthritis models. Additionally, this mechanism boosts BMP-2, TSG-6, and PRG-4 gene expression, which improves cartilage matrix formation (Sekiya et al., 2021). In addition, a Phase I non-randomized trial administered three injections of human placenta-derived mesenchymal stem cells (hP-MSCs) combined with hyaluronic acid (HA) to patients with osteoarthritis (OA) (Table 3). Compared with the control group, the MSC group showed significant improvements in the Western Ontario and McMaster Universities Osteoarthritis Index (WOMAC) and Visual Analogue Scale (VAS) at 6 and 12 months, along with a significant reduction in interleukin-2 (IL-2) expression, suggesting that hP-MSCs have anti-inflammatory effects (Holiuk et al., 2025). Mesenchymal stem cells, which are progenitors to osteoblasts, can develop into osteoblasts in response to environmental and genetic influences. Anti-inflammatory substances like IL-10, which can prevent osteoclast formation and hence shield the subchondral matrix, can be secreted by mesenchymal stem cells. Conversely, pro-inflammatory cytokines IL-6 and IL-1 work in concert to increase osteoclast activity while inhibiting osteoblast activation, which results in the breakdown of bone (Dalle Carbonare et al., 2025). This demonstrates how mesenchymal stem cells contribute to the preservation of subchondral bone homeostasis *via* the “immune bone metabolism” axis.

**TABLE 3:** Summary of representative clinical trials on targeted therapies for osteoarthritis.

Medication/Intervention	Targets/Mechanisms	Pilot phase	Key findings	References
hP-MSCs	—	Phase I non-randomized trial	Improvements in WOMAC and VAS scores; reduced IL-2 expression	Holiuk et al. (2025)
Metformin	Reduce levels of COMP, CTX-1, and IL-1β	Clinical trials	Relieve pain in patients with osteoarthritis who are overweight or obese; inhibit cartilage degeneration and inflammation	Pan et al. (2025), Aiad et al. (2024)
Dexamethasone Sodium Phosphate Compound Liposomes TLC599	Hydrophobic structures bind to synovial fluid; smaller particle sizes are cleared by lymphatic vessels	Phase II clinical trial	Pain relief	Mehta et al. (2021)
FX006: A Micron-Sized PLGA Intra-articular Sustained-Release Formulation of Trichloroacetic Acid	—	Phase III clinical trial	Reduction in WOMAC scores and average daily pain scores	Mehta et al. (2021), Kraus et al. (2018)
Tissue plasminogen activator inhibitor MIV-711	Inhibit bone resorption	Phase IIa Trial Phase I Safety Trial	Improvement in WOMAC pain scores	Brandt et al. (2025)

In conclusion, osteoblasts play a critical role in the development of osteoarthritis. Once thought to be a localized condition brought on by cartilage deterioration, osteoarthritis is now understood to be a systemic condition affecting the entire joint. Among these, aberrant cancellous bone turnover has emerged as a crucial element in controlling the advancement of osteoarthritis. As a result, the osteoblast-chondrocyte crosstalk theory integrates OA with biochemical and biomechanical elements, offering a novel approach for the targeted administration of anti-resorptive medications on osteoblasts in the future to delay the progression of OA.

### 2.3 Cross-talk between immune cells and bone marrow cells

**FIGURE 2:**
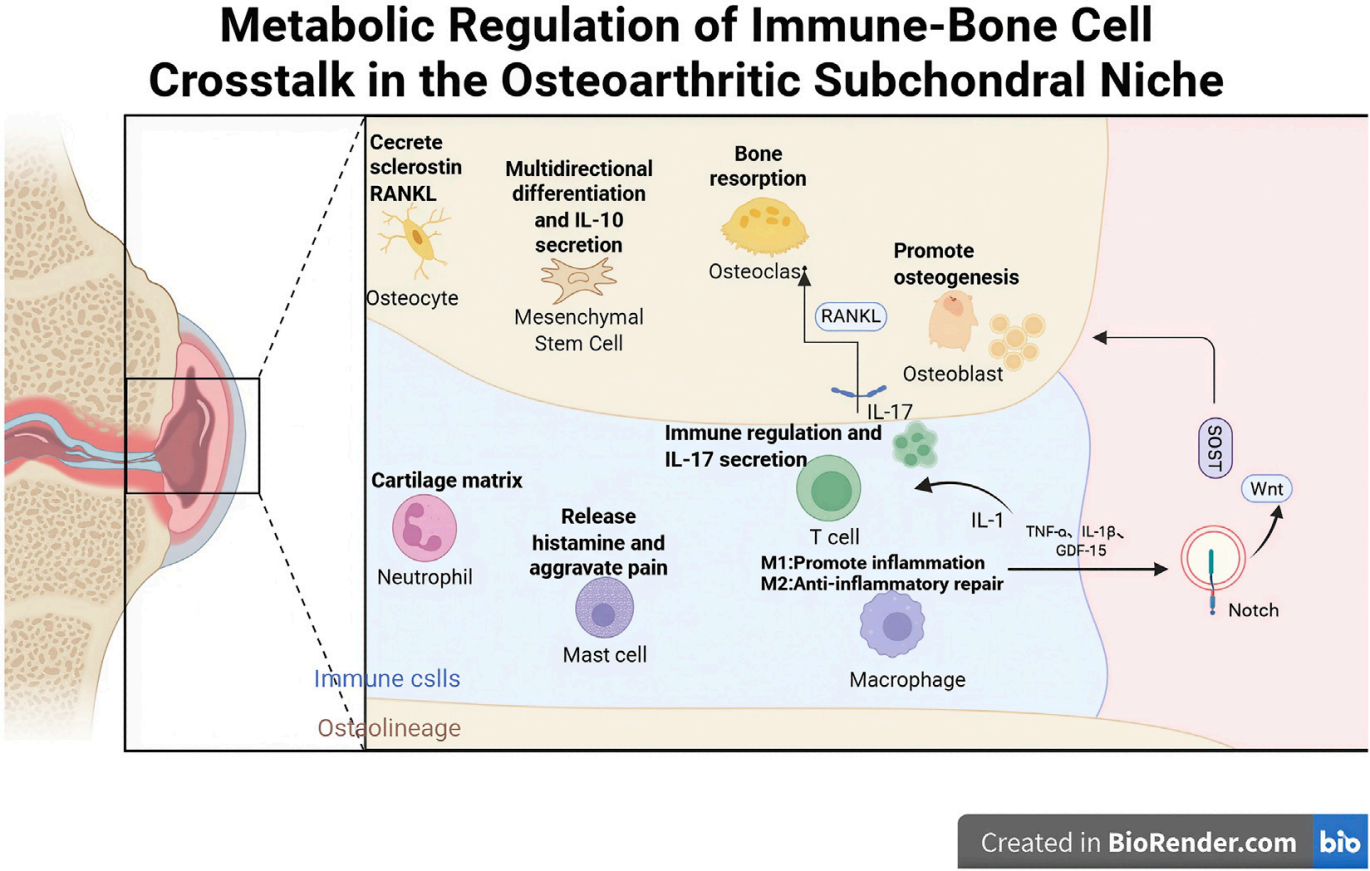
Mechanism of bone metabolic imbalance mediated by M1 macrophages in the subchondral bone microenvironment: promotion of osteoclastogenesis *via* the RANKL pathway and inhibition of osteogenesis *via* the Wnt pathway. Major cells and their functions in the subchondral bone microenvironment; Bone metabolic imbalance driven by macrophages in osteoarthritis. TNF-α and IL-1 released by M1 macrophages drive RANKL expression, facilitating osteoclast formation and activation. Meanwhile, inflammatory mediators like IL-1β and TNF-α secreted by M1 macrophages suppress the Notch pathways in osteoblasts and regulate key osteogenic factors (e.g., SOST) *via* the Wnt pathway, thereby inhibiting bone formation (TNF-α, Tumor Necrosis Factor-alpha; IL-1, Interleukin-1; RANKL, Receptor Activator of Nuclear Factor-κB Ligand; IL-1β, Interleukin-1 Beta; SOST, Sclerostin).

Immune cells and osteoclasts engage in tight functional cross-regulation within the OA pathological milieu through overlapping signaling pathways, metabolic product exchange, and inflammatory mediator release (Figure 2). Because immunometabolism blurs the conventional line between inflammation and metabolism, it offers a crucial perspective for improving understanding of the pathophysiology of osteoarthritis and creating novel treatment approaches.

Maintaining bone health requires a balance between macrophages and osteoblasts, but research on this relationship and its mechanism is currently lacking in the particular pathological context of inflammatory bone remodeling (Wang S. et al., 2022). Tumor necrosis factor-α (TNF-α) has been proven in earlier research to suppress Notch-1 signaling in skeletal muscle cells. Thus, the Notch signaling pathway in osteocytes may likewise be inhibited by TNF-α released by M1 macrophages (Acharyya et al., 2010). Early osteoblast differentiation may be encouraged by inhibiting the Notch signaling system, which may also release the antagonistic influence on the Wnt pathway (Wang S. et al., 2022; Shao et al., 2018). Notch was rendered inactive by the activation of the Wnt, MAPK, and mTOR signaling pathways by IL-1β and TNF-α, respectively. This implies that osteoblasts’ Notch signaling system can be inhibited by M1 macrophages through the induction of inflammatory factor production. According to recent research, the Wnt signaling system controls osteosclerostin (SOST), a crucial inhibitor of osteogenic development. This discovery is related to M1 macrophages’ suppression of the Notch signaling pathway. Thus, aberrant osteogenic differentiation in the early stages of osteoarthritis may be primarily caused by the inflammatory milieu created by M1 macrophages and osteoblasts (Wang S. et al., 2022).

The primary cells involved in bone resorption are osteoclasts, which are descended from the monocyte/macrophage lineage (Ohori et al., 2020; Teitelbaum, 2000). By decreasing the quantity of tartrate-resistant acid phosphatase (TRAP)-positive cells, suppressing the expression of the trap gene, and decreasing the size of resorption pits, IL-33 dramatically reduced TNF-α-induced osteoclast production and bone loss activity, according to *in vitro* investigations (Ohori et al., 2020). Additionally, aberrant osteoclast activation is linked to subchondral bone loss in osteoarthritis, and synovial macrophages may be crucial in this process. Research has demonstrated that CD14^+^ macrophages in the synovial fluid of individuals with osteoarthritis can directly develop into active osteoclasts with bone-resorptive capability in an inflammatory milieu marked by RANKL, IL-1α, and TNF-α (Zhao et al., 2023; Adamopoulos et al., 2006).

In osteoarthritis, the immune environment of the synovium includes a network of interactions between T cells and synovial macrophages in addition to an increase in CD4^+^ T cells (Zhao et al., 2023). Senescent cells (SNCs) control the development of primary T cells into Th17 or Th1 phenotypes by sensing transforming growth factor-β (TGF-β), and *in vitro* tests demonstrated that Th17 cells may cause fibroblast senescence (Faust et al., 2020). A cytokine produced by macrophages called B cell activating factor (BAFF) can stimulate pro-inflammatory Th1 and Th17 cell responses while blocking Th2-mediated anti-inflammatory responses. This could support the theory that macrophages and T cells interact (Zhao et al., 2023; Chen et al., 2014). In addition to directly differentiating into osteoclasts, macrophages can also indirectly increase osteoclast activation by initiating a cascade that involves synovial fibroblasts and CD4+T cells. In particular, Th17 cell differentiation can be induced by TNF-α and IL-1 produced from macrophages. These Th17 cells generate IL-17, which in turn stimulates the development and activation of osteoclast precursors by causing synovial fibroblasts to express RANKL at a high level (Zhao et al., 2023; Takayanagi, 2012). According to recent research, the lack of ubiquitin carboxy-terminal hydrolase L1 (UCHL1) in osteoclast precursors can exacerbate aberrant bone remodeling and subchondral osteoclast production. Conversely, adeno-associated virus 9-mediated high expression of uchl1 can successfully slow the advancement of osteoarthritis (Liang et al., 2024).

The primary cause of aberrant bone remodeling and osteoarthritis is undoubtedly an imbalance in signal transmission between immune cells and osteoblasts. Thus, uncovering this intricate network will offer a fresh target for osteoarthritis immunotherapy.

## 3 Metabolic reprogramming of the subchondral bone microenvironment

### 3.1 Energy metabolism disorder

**FIGURE 3:**
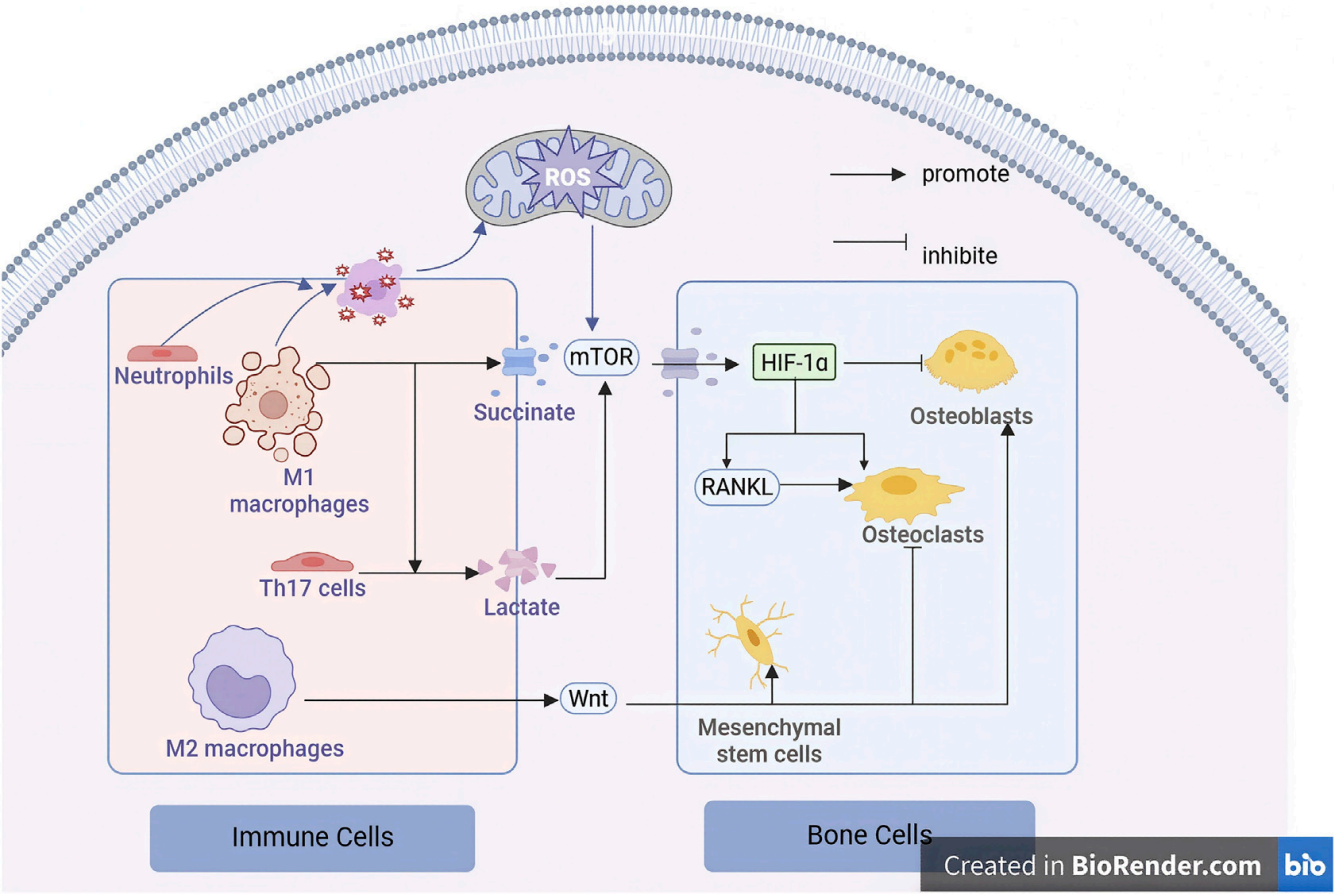
Schematic diagram illustrating the immunometabolic regulation of bone homeostasis by immune cells: Neutrophils, M1 macrophages, and Th17 cells, as pro-inflammatory immune cells, produce key metabolites including succinate, lactate, and reactive oxygen species (ROS). These metabolites converge on and activate the mTOR signaling pathway, leading to the activation of HIF-1α. Activated HIF-1α inhibits osteoblast function while upregulating RANKL expression to promote osteoclast formation and bone resorption. In contrast, M2 macrophages activate the Wnt/β-catenin pathway in mesenchymal stem cells, promoting osteoblast differentiation and bone formation.

One of the fundamental pathogenic mechanisms that propels osteoarthritis forward is energy metabolism abnormalities (Figure 3). Anaerobic glycolysis is the primary energy source for chondrocytes in healthy bones. But in the pathological condition of osteoarthritis, cell metabolic homeostasis is upset, which leads to metabolic reprogramming that exacerbates the inflammatory response and ultimately degrades cartilage (Liao et al., 2024).

The metabolism of glucose plays a major role in the pathogenesis of osteoarthritis. For example, when the Wnt3a protein binds to the LRP5 receptor, it activates the downstream mammalian target of rapamycin complex 2 (mTORC2) pathway, which not only speeds up osteoblast activation (Esen et al., 2013), but also plays a crucial role in the pathophysiology of osteoarthritis. For instance, the Wnt3a protein binds to the LRP5 receptor to initiate the downstream mammalian target of rapamycin complex 2 (mTORC2) pathway, which not only accelerates osteoblast activation but also alters chondrocyte metabolism, initiates the Warburg effect, and prioritizes glycolysis. Furthermore, it has been found that nitric oxide (NO) plays complex dual roles in osteoarthritis. In addition to collaborating with osteoblasts to produce glutathione to eliminate oxidative damage caused by copper ions, it can, in some circumstances, start the Warburg effect, which encourages metabolic reprogramming and bone repair (Zhou et al., 2026).

Hypoxia inducible factor-1α (HIF-1α) is a key element for chondrocytes to adapt to hypoxic metabolic environment (Zhang et al., 2025a). Articular cartilage integrity relies on HIF-1α stability in a hypoxic environment. Destruction of this environment will lead to HIF-1α dysfunction, which brings degenerative changes to chondrocytes (Zhang et al., 2023b). In addition, HIF-1α adjusts cell survival under hypoxic situations by coordinating anaerobic glycolysis and mitosis (Zhang et al., 2023b; Li et al., 2021). Thus, HIF-1α′s primary role in arthritic hypoxic states is to activate the glycolytic pathway, which facilitates the transition from aerobic to anaerobic metabolism. Research has shown that oxidative stress causes hypoxia inducible factor-1α (HIF-1α) to be upregulated in the early stages of osteoarthritis (OA). Later on, inflammation and other variables will accelerate the degradation of HIF-1α, which will impede the glycolysis pathway and accelerate the aging and death of chondrocytes (Zhang et al., 2025a). For progressive osteoarthritis, anti-osteoclast drugs cannot turn back the hypoxic environment destroyed by H-type angiogenesis. At present, maintaining hypoxic environment and stabilizing HIF-1α expression is extremely important; it can not only prevent being restricted by subchondral bone changes but also effectively ease disease progression (Zhang et al., 2023b). In addition, HIF-1α promotes glycolysis by increasing expression of inositol 3-phosphate-dependent protein kinase 1 (PDK1), which regulates glucose metabolism and participates in keeping cartilage matrix homeostasis (Li et al., 2021; Zhang et al., 2023b). In conclusion, hypoxia-inducible factor-1α dysfunction is a core factor in osteoarthritis cartilage degeneration.

Another characteristic of osteoarthritis is an imbalance in the energy supply of chondrocytes. Under normal circumstances, chondrocytes can obtain energy through glycolysis and oxidative phosphorylation. However, oxidative phosphorylation is harmed and glycolysis is unnaturally boosted when osteoarthritis advances. Thus, this imbalance serves as the primary link between cell damage, metabolic malfunction, and osteoarthritis (Arra et al., 2020; Han et al., 2025). One important enzyme that connects two metabolic pathways is pyruvate dehydrogenase (PDH), which allows pyruvate from glycolysis to enter mitochondria and begin oxidative phosphorylation (Han et al., 2025; Li et al., 2022b; Jiang et al., 2025). PDK2 inhibits PDH, which limits oxidative phosphorylation (OXPHOS) and increases glycolysis. Research has shown that chondrocytes in the PDK2 knockout (pdk2ko) mice model exhibit increased oxidative phosphorylation (OXPHOS), decreased generation of reactive oxygen species (ROS), and decreased oxidative DNA damage; thus, metabolic imbalance is improved (Han et al., 2025). Therefore, a key treatment approach for metabolic reprogramming is to reverse energy metabolism from aberrant glycolysis to normal oxidative phosphorylation through targeted inhibition of PDK2.

Consequently, glucose metabolic abnormalities can significantly exacerbate osteoarthritis, damage chondrocyte energy metabolism, and degrade the subchondral bone microenvironment. To return aberrant chondrocyte metabolism to a normal state and boost its capacity for self-healing and survival, targeted modulation of chondrocyte metabolic reprogramming is required.

Disorders in lipid metabolism also have a significant role in osteoarthritis, particularly in osteoarthritis associated with obesity. As an active endocrine organ, adipose tissue secretes a variety of adipokines and inflammatory mediators that contribute to the pathophysiology of osteoarthritis (Zhang et al., 2025b). Increased joint mechanical stress is not the only way that obesity affects osteoarthritis. Bone and cartilage deterioration will be accelerated by systemic metabolic problems associated with obesity. Concurrently, this will result in an unequal distribution of joint forces, causing overload in certain places and cartilage degradation. It is important to note that obesity is significantly associated with an increased incidence of non-weight-bearing osteoarthritis; nevertheless, this phenomenon cannot be entirely explained by mechanical stress alone. Thus, research has highlighted the connection between metabolic processes and free access (Batushansky et al., 2022; Zhang et al., 2025b).

Lipid metabolism is also disrupted in cartilage tissue. Changes in chondrocyte phenotype are closely linked to aberrant metabolism of glycerophosphatide and linoleic acid, according to population research. In type IIA, aberrant phospholipase A2 (PLA2) gene expression can disrupt the lipid metabolism system, exacerbating cartilage damage (Wang Y. et al., 2025). Furthermore, intracellular fatty acid buildup and lipid toxicity result from nuclear factor I-A (NFIA) activation in osteoarthritis cartilage, which upsets the equilibrium between acyl-CoA carboxylase (ACACA) and carnitine palmitoyltransferase 2 (CPT2) expression. Endoplasmic reticulum stress, mitochondrial malfunction, and ultimately the disruption of chondrocyte steady-state homeostasis can result from this toxicity (Wang C. et al., 2025).

Macrophage infiltration in adipose tissue during obesity can release inflammatory adipokines including leptin and adiponectin at the systemic level, accelerating the development of osteoarthritis (Nedunchezhiyan et al., 2022). When 11β-hydroxysteroid dehydrogenase 1 (11β-HSD1) is overexpressed in osteoblasts, glucocorticoid (GC) signaling is overactivated, which inhibits early growth response factor 2 (Egr2), disrupts bone glucose metabolism, and damages osteoblast function. In obese mice, the disruption of glucose metabolism and bone loss can be improved by knocking out this enzyme (Zhong et al., 2024). This deepened our understanding of the obesity bone metabolism axis and provided experimental and theoretical basis for new treatment methods.

Bone is not only a supporting organ, but also an important endocrine organ, which regulates the body’s energy metabolism through secreting various factors. Previous studies have shown that osteopontin secreted by osteoblasts is a key regulator of bone-mediated systemic metabolism (Baroi et al., 2024). Recent studies have shown that peroxisome proliferator-activated receptor γ (PPAR γ) has central role in osteoblasts, regulating lipid, immune, and glucose metabolism. In the osteoblast-specific PPARγ knockout (γOTKO) mouse model, researchers observed increased bone mass and decreased bone marrow cell count, which is consistent with overexpression of the Wnt signaling pathway and enhanced osteoblast proliferation (Baroi et al., 2021). Importantly, this pathway has been proved to be independent of osteopontin, revealing a mechanism that osteocytes regulate systemic metabolism through non-osteopontin-dependent pathways (Baroi et al., 2024). This innovative discovery reveals a new function of PPARG in bone cells and provides a new angle on bone as an endocrine organ. This study revealed two mechanisms, osteopontin-dependent and osteopontin-independent, which cooperatively regulate bone and systemic energy metabolism.

In summary, energy metabolism plays a significant role in the development of osteoarthritis by facilitating the metabolism of fats and carbohydrates. At the same time, bone’s endocrine activity allows it to communicate in both directions with systemic metabolism. Therefore, a promising novel treatment approach to restore energy balance in chondrocytes and osteoblasts is targeted metabolic reprogramming, such as blocking PDK2, stabilizing HIF-1α, or disrupting PPARG signaling.

### 3.2 Metabolic abnormalities of bone cells

The skeletal system’s metabolic abnormalities are a result of a synergistic imbalance between osteoblasts, osteoclasts, and chondrocytes rather than a single cell type disease. Together, these cells’ metabolic reprogramming upsets the steady-state balance of bone remodeling and becomes a major factor.

The metabolism of osteoblasts is highly adaptable to many pathological stressors. According to research, 13 peptide (ADP-1) promotes osteoblast development by activating the lipofuscin receptor, which increases mitochondrial biogenesis and oxidative phosphorylation (OXPHOS). Simultaneously, it inhibits osteoclast activity by blocking the synthesis of RANKL. In order to promote bone mass and strength in ovariectomized mice, ADP-1 can balance bone remodeling by encouraging bone formation and preventing bone resorption (Rajput et al., 2024).

Increased IL-1β expression can promote glycolysis and oxidative phosphorylation (OXPHOS) in osteoblast precursors (MC3T3-E1) in the inflammatory milieu of subchondral bone. Osteopontin (OPN) and alkaline phosphatase (ALP) are also upregulated as a result of this metabolic activity, which encourages osteoblast development. This exacerbates the degenerative course of osteoarthritis and results in subchondral bone sclerosis (Ying et al., 2023). Previous studies have indicated that malate-aspartate transport system (MAS) participates in coordinating mitochondrial function and glycolysis in skeletal muscle cells (Lee et al., 2020). According to recent studies, the loss of mitochondrial transcription factor A (TFAM) in skeletal muscle cells causes an imbalance in mitochondrial acidification (MAS) and oxidative phosphorylation (OXPHOS), which in turn sets off a metabolic compensatory remodeling mechanism. This process promotes the lactic acid fermentation pathway rather than inhibiting glycolysis. In order to maintain the nicotinamide adenine dinucleotide (NAD+) cycle and respond to the energy crisis, it simultaneously couples the glutamine reduction and carboxylation reaction (Khan et al., 2024). This metabolizing activity, which includes HIF-1α-mediated enhanced glycolysis under hypoxic conditions, can compensate for oxidative phosphorylation defects, thus maintaining bone homeostasis (Khan et al., 2024).

Osteoclasts’ maturation and biological characteristics highly depend on metabolic reprogramming, which involves interaction between glycolysis and mitochondrial oxidative phosphorylation (Arnett and Orriss, 2018; Deng et al., 2024). One important mechanism controlling osteoclasts’ involvement in oxidative phosphorylation and glycolysis is osteoclast-specific small GTPase (Rheb1). Research has shown that Rheb1-driven oxidative phosphorylation, rather than glycolysis, is crucial for the production of cat protease K (CTSK), which is required for osteoclast activation. Acetyl coenzyme A expression decreased in Rheb1 mutant mice due to mitochondrial malfunction, which subsequently reduced the quantity of CTSK protein and considerably reduced the capacity of osteoclasts to resorb bone (Deng et al., 2024).

However, osteoclasts and their precursors (OCPs) have undergone a fundamental change in energy metabolism in the inflammatory state of rheumatoid arthritis (RA), preferring glycolysis over oxidative phosphorylation (OXPHOS). This means that while reducing oxidative phosphorylation will increase the number of osteoclasts, inhibiting glycolysis during early differentiation will prevent their development. This highlights the crucial role that glycolysis plays in inflammation-induced bone resorption (Kachler et al., 2024; Srivastava et al., 2022). Second, itaconic acid (4-OI) can effectively inhibit the Acod1 gene deletion that can exacerbate osteoclast development and bone loss in arthritis. By suppressing succinate dehydrogenase (SDH), the Acod1 gene prevents osteoclast development and bone loss by obstructing the reactive oxygen species hypoxia-inducible factor 1α (ROS-HIF1α) signal axis and its subsequent glycolytic pathway (Kachler et al., 2024).

In conclusion, one major factor hastening the onset of osteoarthritis (OA) is aberrant bone cell metabolism. Chondrocyte metabolic abnormalities in the synthesis and degradation processes are directly caused when the equilibrium between osteocyte-mediated bone production and osteoclast-mediated bone resorption is upset. This ultimately results in pathological diseases such articular cartilage degradation by upsetting the subchondral bone microenvironment. In order to enable tailored treatment of osteoarthritis, it is crucial to investigate aberrant bone metabolism underlying molecular processes.

### 3.3 Metabolites as signaling molecules

Unusual chondrocyte metabolism leads to an excessive accumulation of metabolic byproducts in osteoarthritis. These metabolites serve purposes beyond those of traditional metabolism. They are important signaling molecules that play a major role in the development of osteoarthritis by causing pain, driving cartilage degradation, and deeply regulating inflammatory responses.

In addition to being the result of glycolysis, lactic acid is a multipurpose signal molecule that plays a crucial role in connecting metabolic irregularities and the development of osteoarthritis. Research shows that lactic acid can increase the production of NADPH Oxidase 4 (NOX4) by activating the downstream PI3K/Akt signaling cascade through its receptor nicotinamide adenine dinucleotide phosphate (NADPH). Reactive oxygen species (ROS) build up as a result, eventually damaging chondrocytes and exacerbating inflammation and metabolism. Targeted elimination of NOX4 and ROS was shown *in vivo* to prevent lactic acid from harming joint structure (Huang et al., 2023). Second, lactic acid buildup was brought on and maintained throughout the conversion of pyruvate to lactic acid in the chondrocyte matrix. This acidic environment accelerated the breakdown of cartilage and reduced the matrix’s chondrogenic capacity (Zheng et al., 2021). More significantly, tissue acidification and lactic acid buildup are two of the primary causes and maintainers of pain, which has a direct negative influence on patients’ quality of life (Zheng et al., 2021; Wen et al., 2023).

The role of protein post-translational modification, particularly succinylation, in diseases of bone metabolism has drawn increasing interest. The tricarboxylic acid cycle’s (TCA cycle) efficiency and ATP production are impacted by succinylation, which also influences osteoblast proliferation, differentiation, and bone matrix synthesis (Kong et al., 2026). Succinylation omics in patients with osteoporosis (OP) provides preliminary support for this opinion, and it also aligns with the following finding: In rheumatoid arthritis (RA), the desuccinylase sirt5 maintains mitochondrial homeostasis, which regulates cellular activity (Kong et al., 2026; Mao et al., 2023).

Oxidative phosphorylation (OXPHOS) activity is compromised in the pathological condition of osteoarthritis, which results in an excessive build-up of reactive oxygen species (ROS) and reactive nitrogen species (RNS) as well as insufficient ATP synthesis. This creates a vicious cycle of oxidative stress and energy depletion, which ultimately exacerbates the permanent damage to chondrocytes (Guan et al., 2022; Xiang et al., 2024). Novel approaches to nanoenzyme treatment have demonstrated significant promise in resolving this issue. According to research, when exposed to infrared light, g-C3N4-loaded platinum single-atom (PtSA/C3N4) nanozymes collectively reduce ROS/RNS expression levels and increase the expression of ATP synthase and respiratory chain complex I. This repairs the inflammatory milieu of chondrocytes by lowering oxidative stress and inflammatory responses (Xiang et al., 2024).

To sum up, metabolites that play important signaling functions in osteoarthritis include lactic acid, succinylation changes, and reactive oxygen or nitrogen species. Clarifying the mechanisms of action of these metabolites not only provides a scientific foundation for comprehending the inflammation-metabolism-bone degradation axis, but it also enables the targeting of metabolic signaling molecules for the therapy of osteoarthritis. Therefore, more investigation into the interplay between metabolic signaling networks and osteoblasts will facilitate the creation of more focused therapeutic drugs.

## 4 Therapeutic potential targeting immune metabolism

### 4.1 Regulating the metabolism of immune cells

The development of OA is closely linked to aberrant immune cell metabolism. The main strategy in bone-joint immunometabolism research is to modify the metabolic status of immune cells, such as macrophages, in order to restore the subchondral bone microenvironment and reverse the pro-inflammatory state (Table 4). As a traditional blood glucose-lowering medication, metformin stabilizes blood glucose levels by targeting the intestines, liver, and peripheral tissues to slow down absorption, enhance glucose uptake and utilization, and limit gluconeogenesis (Zheng et al., 2023). A study has shown that metformin can significantly reduce pain in overweight or obese patients with osteoarthritis (6-month VAS score difference between groups: −11.4 mm, P = 0.01, effect size 0.43). The findings support its efficacy; however, due to the limited sample size, further validation in larger trials is needed (Pan et al., 2025). The current challenge in osteoarthritis (OA) treatment strategies is the need to both relieve pain and halt or delay the degeneration of articular cartilage and changes in subchondral bone. A new clinical trial has shown that metformin adjunctive therapy can reduce serum levels of COMP, CTX-1, and IL-1β in patients with obesity-related OA, suggesting that it can inhibit cartilage degeneration and inflammation. Meanwhile, improvements in WOMAC scores confirm its clinical efficacy, and its mechanism of action may involve anti-inflammatory effects, chondroprotection, and the regulation of bone remodeling and metabolism (Aiad et al., 2024). This suggests that metformin may reduce the progression of OA, according to numerous studies (van den Bosch, 2021). On the one hand, metformin slows down cartilage degradation by preventing M1 macrophages from secreting proinflammatory cytokines and inhibiting their inflammatory activation of chondrocytes in conditioned media. Additionally, it inhibits the migration of macrophages induced by IL-1β. Thus, by decreasing M1 macrophage infiltration in the synovium, this strategy can successfully postpone cartilage degradation (Zheng et al., 2023). On the other hand, related research has found that the initial occurrence of OA has a close connection with ferroptosis, which is a new type of cell death taking place in chondrocytes (Martin-Sanchez et al., 2018; Zou Z. et al., 2025). In the pathogenic settings of OA, macrophages release inflammatory mediators that damage the subchondral matrix and kill chondrocytes (Carlson et al., 2021). Ferroptotic chondrocytes express more macrophage chemotactic factor (CCL2), which causes widespread macrophage migration and intensifies the inflammatory response. Metformin inhibits the recruitment of macrophages and stops CCL2 release, which effectively reduces the inflammatory response brought on by ferroptosis (Zou Z. et al., 2025). Lastly, by focusing on iron death and ending the vicious cycle of osteoarthritis through the immunometabolic route, metformin expands its conventional therapeutic role.

**TABLE 4:** Primary drugs, targets, and mechanisms of action for osteoarthritis treatment.

Treatment strategy categories	Representative targets/Drugs	Mechanism of action	References
Regulating the metabolism of immune cells	Metformin	M1 macrophages ↓→ pro-inflammatory cytokines↓, chondrocyte ferroptosis and inflammation↓	Zheng et al. (2023), Zou et al. (2025b)
Regulating bone cell metabolism	PDK2	PDK2 ↓→ chondrocyte OXPHOS → senescence,oxidative stress↓	De Ceuninck et al. (2004), Han et al. (2025)
	HIF-1α	chondrocyte autophagy↑; oxidative stress and expression of β-catenin/HIF-2α↑; subchondral bone sclerosis↓	Chen et al. (2025)
Targeting joint metabolic enzymes	lactic acid	c-Fos → PDH/LDH balance↑ → chondrocyte proliferation and collagen synthesis↑	Matsuoka et al. (2023)
		PI3K/Akt signaling pathway → reactive oxygen species (ROS) and cellular damage; NOX4 inhibitor →damage↓	Huang et al. (2023)
	succinic acid	SDH → block HIF-1α/VEGF pro-angiogenic axis	Li et al. (2018)
		Targeting G protein-coupled receptors (GPCRs)	Saraiva et al. (2018)
		Gabapentin → ABAT ↓ → mitochondrial respiratory dysfunction and articular cartilage degradation	Shen et al. (2019)
Nanotechnology Targeted Delivery System	Rapamycin	Nanoparticles release their loaded astaxanthin (AST) + rapamycin (RAPA)→ M1 macrophage polarization	Li et al. (2024)
		macrophage mTORC1 pathway and enzymatic oxidative stress↓ → cGAS-STING signaling pathway↓	Qi et al. (2024)
	Polylactic-Polyhydroxyacetic Acid (PLGA) Copolymer Microparticles	Targeted Delivery of Magnesium Ions (Mg^2+^) to Articular Cartilage	Zheng et al. (2024)

Hence, it has now become a promising treatment method for osteoarthritis, which inhibits inflammation and pushes forward cartilage repair by precisely regulating the metabolic network of immune cells inside the articular cavity. Whether it is a new nanodrug based on mTOR inhibitors or a conversion drug like metformin, this all shows the very great potential of an immunometabolic therapy strategy.

### 4.2 Regulating bone cell metabolism

In the cartilage microenvironment, the metabolic balance of osteoblasts gets destroyed, thus causing abnormal subchondral bone remodeling and speeding up cartilage degradation. Therefore, intervention on osteoblast metabolism has become a frontier treatment strategy to reverse osteoarthritis’s pathological progression.

One of the main tactics is to correct the imbalance in chondrocyte energy metabolism. Research has shown that fibroblast-like synoviocytes that are positive for thymus antigen-1 in osteoarthritis change their metabolism to glycolysis by increasing the expression of pyruvate dehydrogenase kinase (Damerau et al., 2022). In chondrocytes, this process is also very important. Omics data have demonstrated ongoing upregulation of inflammatory signals in articular cartilage, despite the fact that osteoarthritis was previously thought to be a solely degenerative illness (De Ceuninck et al., 2004). According to recent studies, inhibiting PDK2 expression in pathological settings can reroute chondrocyte metabolism toward the oxidative phosphorylation pathway, reducing oxidative stress and cell aging and ending the vicious cycle of osteoarthritis (De Ceuninck et al., 2004; Han et al., 2025). Therefore, by addressing PDK, chondrocyte metabolic anomalies can be fundamentally corrected, delaying or perhaps stopping the evolution of the disease. This approach is still in the fundamental research stage, though, and more research is needed to confirm its potential for therapeutic transformation.

HIF-1α is a key protective factor for maintaining chondrocyte survival and function in a hypoxic microenvironment (Yao et al., 2023). Its activity and stability are carefully controlled on several levels. For instance, hypoxia-inducible factor-1α and lysine demethylase 5C interact. Cartilage homeostasis can be destroyed by its lack, which can suppress the HIF-1α signaling pathway and cause oxidative stress and mitochondrial metabolic disorders. Conversely, in a mouse model, KDM5C function restoration can successfully slow the advancement of osteoarthritis (Ding et al., 2025). In terms of mechanisms, HIF-1α activation can delay cartilage breakdown by promoting autophagy and lowering oxidative stress. Furthermore, its protective role is linked to the regulation of β-Catenin and HIF-2α expression, suggesting that HIF-1α activation has a multifaceted protective role by protecting cartilage by lowering subchondral bone sclerosis (Chen et al., 2025). The crucial physiological state for sustaining the activity and function of hypoxia-inducible factor-1α is the long-term maintained moderately hypoxic environment of articular cartilage (Yao et al., 2023; Yang et al., 2010). Therefore, people might anticipate improving the cartilage microenvironment and delaying the course of osteoarthritis by controlling HIF-1α activity in conjunction with therapeutic strategies that target other signaling pathways. However, there are still several issues with associated therapeutic approaches in the clinical transformation process, including safety, tissue targeting, and long-term efficacy, which require more investigation to resolve.

In summary, targeted bone metabolism corrects chondrocyte metabolic anomalies, promotes anabolism, and inhibits catabolism by controlling important metabolic enzymes (like PDK) and signaling pathways (like HIF-1α). This topic offers a viable path for the development of novel treatments for osteoarthritis by promoting metabolic reprogramming to restore the energy equilibrium of joint tissue.

### 4.3 Targeting joint metabolic enzymes

Osteoarthritis is primarily caused by abnormal activity of joint metabolic enzymes. Targeted modulation of these enzymes has emerged as a key area of study for the treatment of osteoarthritis to address the accumulation of metabolic byproducts and signal transduction disorders. Chondrocytes’ energy metabolism balance depends on the exact control of important metabolic enzymes. According to studies, chondrocyte deletion of the c-Fos gene can cause an imbalance in the activity of aerobic oxidative pyruvate dehydrogenase (PDH) and lactate dehydrogenase (LDH) by upregulating hypoxia-inducible factor-1 α (HIF-1 α). Collagen synthesis and cell proliferation will ultimately be harmed by this imbalance. Therefore, recovering the PDH/LDH activity balance by focusing on the metabolic pathway controlled by c-Fos has emerged as a successful tactic to stop the pathological evolution of osteoarthritis (Matsuoka et al., 2023).

As a byproduct of glycolysis, lactic acid has a complicated dual role in osteoarthritis. It functions as both an energy substrate and a signaling molecule by upregulating NADPH Oxidase 4 (NOX4) and activating the phosphatidylinositol 3-kinase (PI3K)/protein kinase B (Akt) signaling axis. It simultaneously supplies NADPH through metabolic reprogramming. Together, these two mechanisms create a vicious cycle that damages cells and produces reactive oxygen species (ROS). Pretreatment with NOX4 inhibitors can lessen this harm (Huang et al., 2023). In addition to classical targeted regulatory pathways, recent studies have identified novel potential therapeutic targets associated with lactate metabolism. Under physiological conditions, UCHL1 knockout does not affect the osteoarthritis phenotype in mice. However, in DMM-induced OA models and in patients, UCHL1 expression in subchondral bone is elevated. Although this is associated with increased osteoclast activity, UCHL1 itself inhibits osteoclast formation. This suggests that the body attempts to compensate for excessive bone resorption by upregulating UCHL1; however, this compensatory mechanism is insufficient under (Liang et al., 2024; Feng et al., 2023). Therefore, enhancing UCHL1 function may restore bone metabolic balance, reduce bone resorption, and thereby slow the progression of OA. Specifically, under pathological conditions, UCHL1 is abnormally upregulated in subchondral osteoclast precursor cells, disrupting the RANKL–UCHL1–sCD13 negative feedback regulatory pathway. This, in turn, promotes excessive proliferation and activation of osteoclasts, inducing pathological resorption of subchondral bone and destruction of trabecular bone structure, thereby accelerating the progression of OA (Liang et al., 2024). Animal studies have demonstrated that conditional knockout of UCHL1 in osteoclast precursor cells significantly inhibits abnormal bone remodeling and delays joint degeneration (Feng et al., 2023), However, small-molecule inhibition strategies targeting UCHL1 are currently still in the preclinical animal research phase. By controlling the subchondral bone microenvironment and reducing underlying inflammatory mechanisms, lactic acid offers a novel approach for comprehensive therapy of osteoarthritis, even though further research is required to support its use.

An important signal molecule in the inflammatory process is succinic acid, a metabolite that is released into the cytoplasm from the mitochondrial tricarboxylic acid cycle. Metabolites generated from macrophages control inflammatory catabolism by altering the functional state of stromal cells (Henry and O'Neill, 2025). Succinic acid can maintain the hypoxia-inducible factor-1α (HIF-1α) protein within macrophages in a joint hypoxic milieu, which might exacerbate the inflammatory state of arthritis by promoting the release of pro-inflammatory factors like interleukin-1β (IL-1β) (Henry and O'Neill, 2025; Tannahill et al., 2013). In addition, extracellular succinic acid mediates multiple biological effects by activating G protein-coupled receptor 1 (SUCNR1, also known as GPR91) on the cell membrane. While previous studies generally regarded GPR91 as a pro-inflammatory regulatory molecule, recent research has shown that this receptor exhibits both pro-inflammatory and anti-inflammatory regulatory activities depending on the microenvironment and cell subtype (Huang H. et al., 2024). Pro-inflammatory macrophages produce succinic acid, but the corresponding receptor, SUCNR1, is preferentially expressed on the cell surface of anti-inflammatory macrophages. Extracellular succinic acid binds to SUCNR1 *via* the Gq and PLC signaling pathways, inducing mesenchymal stem cells to secrete PGE2, which in turn promotes the transition of macrophages from a pro-inflammatory phenotype to an anti-inflammatory phenotype (Trauelsen et al., 2021; Yuan et al., 2022). Consequently, tailored succinic acid offers a novel method of treating osteoarthritis. Studies have demonstrated that dihydromaleate diesterase (DMM) inhibits the HIF-1α/VEGF angiogenic axis *via* transforming fibroblast-like synoviocytes (FLS) and competitively inhibiting succinate dehydrogenase (SDH) (Li et al., 2018). Furthermore, the absence of succinate receptor 1 (sucnr1) can lessen the severity of antigen-induced arthritis, demonstrating the potential benefit of focusing on G protein-coupled receptors (GPCRs) (Saraiva et al., 2018). Succinic anhydride receptor antagonists are a novel therapeutic approach that can successfully prevent U937 cells activated by succinic acid or human synovial fluid from secreting interleukin-1β (Littlewood-Evans et al., 2016). Furthermore, an intermediary in this process is 4-aminobutyric acid aminotransferase (ABAT), an enzyme that catalyzes the conversion of γ-aminobutyric acid to succinic acid. Overexpression of ABAT in chondrocytes can accelerate the deterioration of articular cartilage and enhance mitochondrial respiration. On the other hand, arthritis progression can be stopped by using gabapentin, an inhibitor (Shen et al., 2019). Building on this, new research provides genetic evidence that upregulation of ABAT in chondrocytes leads to chondrocyte catabolism and accelerates the progression of osteoarthritis in mice. Overexpression of ABAT in chondrocytes enhances mitochondrial respiration and leads to increased expression of genes associated with catabolism and hypertrophy. Conversely, inhibition of ABAT attenuates the expression of these genes in IL-1β-treated articular chondrocytes, reduces mitochondrial respiration, and slows the progression of osteoarthritis in mice following MLI surgery (Shen et al., 2019; Shen et al., 2017). In articular cartilage, ABAT is negatively regulated by the epigenetic factor DNMT3B; downregulation of DNMT3B during the progression of osteoarthritis (OA) leads to reduced methylation levels in the ABAT promoter region, resulting in upregulation of ABAT expression. Overexpression of ABAT promotes cartilage matrix degradation by enhancing mitochondrial respiration, whereas treatment with its inhibitor vigabatrin or gene knockdown significantly alleviates pathological changes associated with OA (Shen et al., 2019). However, due to metabolic redundancy and compensatory mechanisms, solely focusing on succinic acid may present difficulties due to the intricate pathophysiological mechanisms of osteoarthritis. Future clinical studies must therefore confirm if succinic acid therapy can continue to be effective without experiencing a significant decline as a result of compensatory adaptation.

Using small-molecule medications to reprogram pathological chondrocyte and immune cell metabolic states and return them from a pro-inflammatory catabolic state to a normal state is, in essence, the core of joint metabolic enzyme treatment strategies. The pathological development of osteoarthritis is intimately linked to the functional imbalance of several metabolic enzymes. These enzymes are essential for the breakdown of cartilage matrix and the activation of the inflammatory response, which offers a rich molecular foundation for the creation of precision enzyme-targeted osteoarthritis treatments.

### 4.4 Emerging therapeutic paradigms: nanotechnology and targeted delivery systems

Due to the medications’ systemic distribution, which results in a limited therapeutic impact and an increased risk of systemic adverse effects, traditional pharmacological therapy for osteoarthritis frequently fails to achieve sufficient local concentration. Because of their superior biocompatibility, biodegradability, and multifunctional drug-loading capacity, nanoparticles (NPS) offer a novel approach to overcoming the therapeutic bottleneck. Many therapeutic medications, including DNA, peptides, and small-molecule compounds, can be efficiently delivered using nanoparticles (Xiao et al., 2022; Liang et al., 2021). Drugs can be wrapped or connected to prevent premature breakdown during delivery by utilizing nanoparticles’ ability to pass through different biological barriers. This clearly improves stability, solubility, and bioavailability (Xiao et al., 2022). The emergence of nanotechnology, particularly the quick development of tailored delivery systems, offers a solution to the problem of osteoarthritis treatment.

Rapamycin targets protein kinase (mTOR), a conserved serine/threonine protein kinase that acts as a core hub to sense internal and external signals. It mainly controls the activation and polarization of immune cells by coordinating key biological processes like cell growth, metabolism, and autophagy (Yao et al., 2023; Klionsky, 2010). In recent years, nanodelivery systems based on rapamycin have received great attention, for they can realize precise targeting, strengthen therapeutic effect, and lower toxicity. Research confirms that nanoparticles (NP@PolyRHAPM)quickly break apart in the highly reactive oxygen species (ROS) environment of M1 macrophages and release astaxanthin (AST) and rapamycin (rapa) that M1 macrophages load. These compounds together remove reactive oxygen species (ROS), enhance autophagy, and inhibit NLRP3 inflammasome activation. This effectively promotes the transformation of macrophages from the pro-inflammatory M1 subtype to the anti-inflammatory M2 subtype, and finally protects articular cartilage (Li et al., 2024). Another study found rapamycin-loaded mesoporous Prussian blue nanoparticles targeting MMP9 (RAPA@MPB-MMP9 NPs). The nanoparticles can double inhibit the activation of the CGA signaling pathway through inhibiting mTORC1 and reducing reactive oxygen species (ROS) activity in macrophages. This repair recovers mitochondrial homeostasis, thus slowing down articular cartilage degradation and providing a new treatment strategy for osteoarthritis (Qi et al., 2024). At present, more and more studies combine traditional Rapa with new nanomaterials, aiming to upgrade from broad-spectrum therapy to precision therapy (Li et al., 2024; Qi et al., 2024). In conclusion, the innovation of rapamycin nanoparticles is to realize targeted drug delivery *via* advanced delivery systems. At the same time, they target inflammatory cells and regulate autophagy, hence synergistically interfering with multiple pathological mechanisms underlying osteoarthritis.

In terms of delivery systems, research has also focused on modulating the joint microenvironment through materials science approaches. For example, researchers have developed a microparticle delivery system using poly (lactic-co-glycolic acid, PLGA) as a matrix, which enables the sustained, targeted release of magnesium ions (Mg^2+^). This system has been shown to improve the pathological condition of subchondral bone, thereby slowing the progression of osteoarthritis (OA), demonstrating the therapeutic potential of combining metabolic intervention with biomaterials (Zheng et al., 2024). For intra-articular administration, TLC599, developed by Taiwan Liposome Co., is a 130-nm-particle-size dexamethasone sodium phosphate-conjugated liposome. Its hydrophobic structure allows it to bind to synovial fluid, while its particle size reduces lymphatic clearance. Meanwhile, a Phase II clinical trial (NCT03005873) confirmed that following a 12 mg intra-articular injection, the drug demonstrated significantly superior pain relief compared to placebo over a 1- to 24-week period (Mehta et al., 2021). In addition, a micron-sized PLGA intra-articular sustained-release formulation (FX006) loaded with trichloroacetic acid has recently been approved for clinical use to alleviate pain caused by osteoarthritis (Kraus et al., 2018). A Phase III clinical trial of FX006 evaluated its efficacy in patients with unilateral knee osteoarthritis. The results confirmed that, compared with saline and standard comparator drugs, it significantly reduced WOMAC scores and average daily pain scores within 24 weeks (Mehta et al., 2021).

**FIGURE 4:**
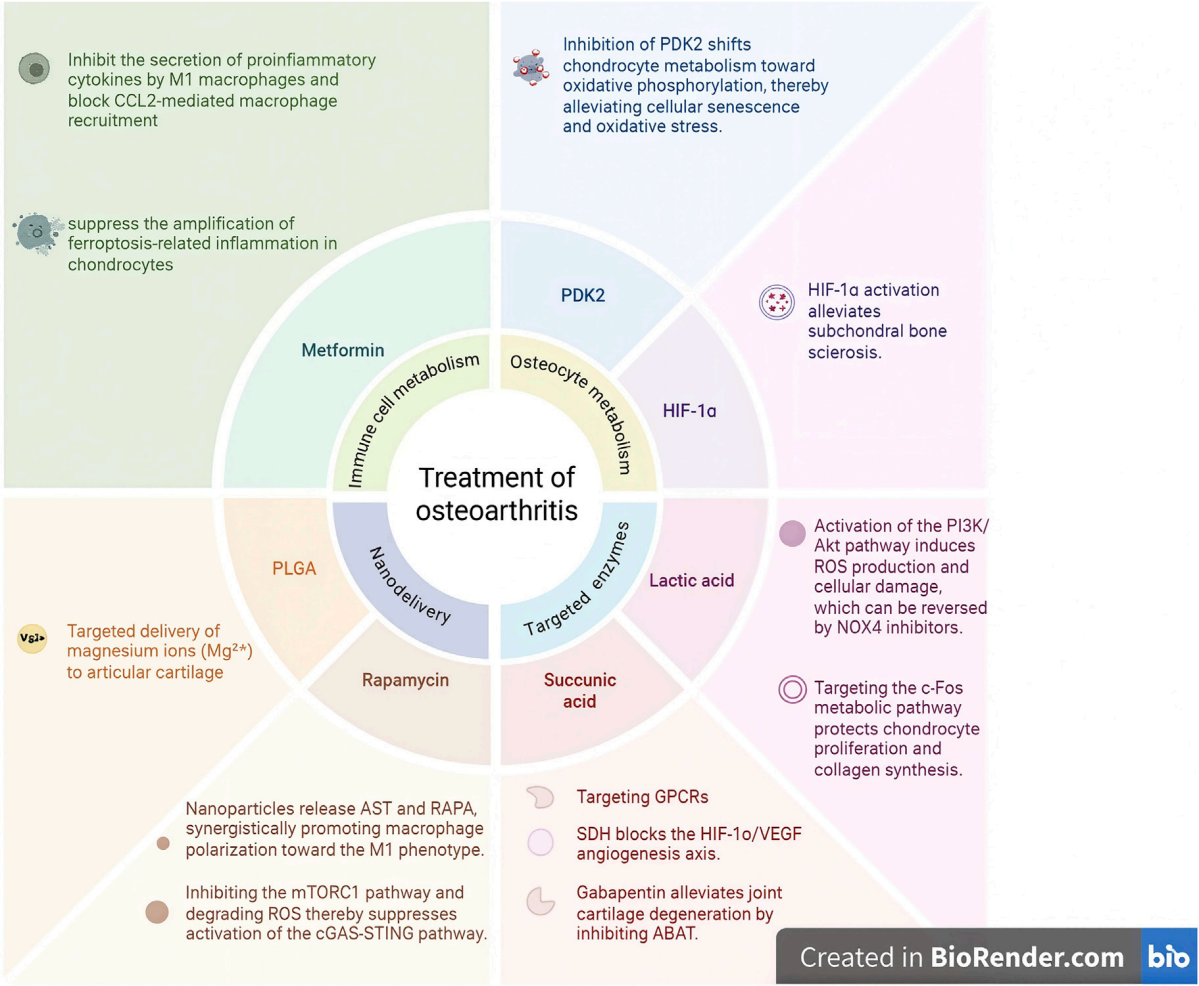
Treatment strategies and mechanisms of action for osteoarthritis (CCL2, C-C Motif Chemokine Ligand 2; PDK2, Pyruvate Dehydrogenase Kinase 2; HIF-1α, Hypoxia-Inducible Factor 1-Alpha; c-Fos, Cellular Fos proto-oncogene; PDH, Pyruvate Dehydrogenase; LDH, Lactate Dehydrogenase; PI3K, Phosphoinositide 3-Kinase; Akt, Protein Kinase B; NOX4, NADPH Oxidase 4; SDH, Succinate Dehydrogenase; VEGF, ascular Endothelial Growth FactorGPCR; ABAT, 4-Aminobutyrate Aminotransferase; RAPA, Rapamycin; cGAS, Cyclic GMP-AMP Synthase; STING, Stimulator of Interferon Genes; PLGA, Poly (lactic-co-glycolic acid; mTORC1, mammalian target of rapamycin complex 1).

In summary, nanotechnology-based targeted therapy represents a significant shift in treatment philosophy as well as technological advancement. The nano-targeted delivery system will open a new chapter in the treatment of osteoarthritis and achieve a shift from conventional palliative care to pinpoint repair thanks to its benefits of high efficiency and precision (Figure 4).

## 5 Challenges and future outlook

### 5.1 Technical challenges

For a long time, osteoarthritis, a common degenerative joint disease, has been regarded as involving features of articular cartilage damage, inflammatory reaction, and metabolic irregularities. At present, immune metabolic adjustment has become a novel intervention target. However, disease heterogeneity and insufficient target specificity bring about great difficulties for clinical treatment. In the future, therefore, we must employ multi-omics analysis to disclose potential mechanisms and optimize treatment strategies, hence providing more precise diagnoses and treatment schemes for patients.

In bone and cartilage disease research and the drug development area, traditional two-dimensional cell culture and animal models have turned into key bottlenecks in translational medicine, because they have huge differences from the human body’s actual pathophysiological environment (Lin et al., 2020; Huang J. et al., 2024). Secondly, the connection part between subchondral bone and cartilage has complex structural properties, so it is hard to get complete tissue samples by using existing minimally invasive biopsy technology. In addition, such operations may disrupt the local microenvironment and cause sample deformation. The appearance of single-cell RNA sequencing (scRNA SEQ) technology has pushed knee osteoarthritis (KOA) research into a new era of high-resolution cell analysis, which systematically unveils its complicated cellular heterogeneity and potential molecular driving mechanisms (Tang et al., 2024; Liu et al., 2025; Gu et al., 2023). Single-cell multiomics technology is deepening our comprehension of osteoarthritis and solving the limitations of transcriptomic research alone (Gu et al., 2023). Although these technologies produce massive data and new understandings for musculoskeletal disease research, the core challenge still remains in how to convert these data into diagnostic tools and effective therapies with clinical value (Rat et al., 2020).

Metabonomics reveals the close relation between osteoarthritis and systemic metabolic disorders by systematically analyzing small-molecule metabolites. Great heterogeneity is the core challenge of osteoarthritis. Due to differences between patient subgroups and chondrocyte subtypes, it is difficult to establish a single-path treatment strategy (Ji et al., 2019; Fan et al., 2024). Metabonomics, combined with single-cell sequencing and other technologies, can precisely classify cell subsets according to their metabolic features. For instance, chondrocytes from patients can be divided into inflammation-related and fibrosis-related subtypes. Based on this, we can use these cell characteristics to carry out hierarchical analysis of patients so as to achieve precision medicine (Fan et al., 2024).

Our knowledge of osteoarthritis has completely changed as a result of the collaborative application of metabonomics and single-cell sequencing techniques. Collaborative studies have demonstrated that osteoarthritis is now a complex process driven by several cell subtypes and associated immunometabolism rather than a single pathogenic process. This offers a fundamental theoretical basis for resolving the osteoarticular cell heterogeneity problem.

### 5.2 Scientific problems

There is a great deal of variation in the pathophysiology of osteoarthritis. Subtype classification based on subchondral bone immunometabolism has become essential to comprehending its pathophysiological causes and advancing targeted therapy as research progresses. Immune cells, such as T cells and macrophages, interact with osteoclasts and osteoblasts in many subtypes and exhibit unique metabolic reprogramming events. Together, these cells create a disordered milieu driven by inflammation and marked by metabolic imbalance. As a result, examining cell contact networks in certain subtypes offers a fresh perspective and possible treatment targets for osteoarthritis treatments.

The initial acute mechanical injury of the joint and the ensuing chronic biomechanical imbalance represent the pathophysiological basis of primary traumatic osteoarthritis (PTOA) (Dilley et al., 2023). Large amounts of oxygen-free radicals can be released following acute trauma due to mitochondrial malfunction; these radicals can trigger an intracellular inflammatory response by activating the mitogen-activated protein kinase (MAPK) pathway and upregulating interleukin-6 (IL-6). In the meantime, this process will promote extracellular matrix disintegration and chondrocyte death, which will ultimately set off a broad inflammatory cascade inside the joint capsule (Dilley et al., 2023; Ayala et al., 2021).

It is well established that systemic low-grade inflammation and localized inflammatory states are closely related to osteoarthritis. This condition closely resembles the low-grade, chronic inflammatory phenotype associated with aging, and inflammatory aging may be a major factor in the development of OA (Attur et al., 2015; Loeser et al., 2016). Joint cell senescence is primarily caused by oxidative stress and age-related mitochondrial failure. By secreting a distinct senescence-associated secretory phenotype (SASP), these senescent cells cause cartilage degradation and increase the incidence of OA (Coryell et al., 2021). New research has found that the progression of osteoarthritis is closely linked to the accumulation of senescent cells in joint tissues; the senescence-associated secretory phenotype (SASP), age-related mitochondrial dysfunction, and oxidative stress collectively contribute to cartilage degeneration (Coryell et al., 2021). Building on these findings, subsequent studies have further revealed a bidirectional regulatory relationship between immune metabolites and cellular senescence. Research indicates that in avascular articular cartilage, chondrocytes primarily rely on anaerobic respiration, and lactate accumulation depends on MCT4 efflux to maintain pH homeostasis (Chen et al., 2026). During the aging process, this metabolic balance is completely disrupted. Aging chondrocytes and the cells present in aged cartilage commonly exhibit mitochondrial dysfunction, which further drives metabolic reprogramming. This leads to increased ROS production and induces compensatory upregulation of glycolysis, ultimately exacerbating lactate accumulation (Chen et al., 2026; Loeser et al., 2014). This creates a vicious cycle: age-related mitochondrial dysfunction drives a metabolic shift toward glycolysis, leading to the accumulation of large amounts of lactate; conversely, elevated lactate levels and an acidified microenvironment further inhibit mitochondrial function, inducing and perpetuating a state of cellular senescence (Huang et al., 2023). Clarifying the precise processes by which senescent cells accelerate the progression of OA remains a major focus and difficulty in current research, even though aging is a major contributing factor to the disease but not its only cause.

Therefore, there are key distinctions between age-related OA and PTOA in terms of subchondral bone immunometabolism. The latter is the outcome of cumulative functional loss brought on by the combined impacts of internal aging and metabolic dysfunction, whereas the former typically includes acute or chronic immunometabolic reprogramming brought on by external mechanical trauma. As a result, the targeted immunometabolic treatments for the two illnesses differ as well: While age-related OA benefits from improved metabolism and senescent cell clearance, PTOA requires potent anti-inflammatory therapy and early immunomodulation to stop the inflammatory cascade. In the future, overcoming the clinical problems of osteoarthritis will require comprehensive patient classification employing multi-omics technologies in conjunction with tailored medicines that target specific disease pathways.

### 5.3 Conceptual limitations and translational bottlenecks in research on immunometabolism and metabolic reprogramming for the treatment of osteoarthritis

Currently, research into the relationship between subchondral bone remodeling and immunometabolic dysregulation in osteoarthritis (OA) is ongoing, gradually revealing the metabolic interactions between various immune cells and osteocytes. However, this field still faces a series of inherent limitations and developmental bottlenecks regarding the completeness of theoretical understanding, the accuracy of conceptual definitions, and the feasibility of translating basic research into clinical applications.

The concept of immunometabolism was introduced more than a decade ago to describe the bidirectional relationship between metabolism and immunity. Early research focused on immune activation in metabolic disorders such as obesity and insulin resistance, providing an initial definition of immunometabolism (Hu et al., 2024). As our understanding of metabolic pathways in immune cells—both in activated and quiescent states—continues to deepen, the definition of immunometabolism has been greatly expanded (Hu et al., 2024; Pearce and Pearce, 2013). Under inflammatory conditions, cells actively undergo metabolic reprogramming to meet changing energy and biosynthetic demands (Wang et al., 2026). Previous studies have largely focused on immune cells as the primary agents of metabolic reprogramming, overlooking the importance of chondrocytes, the extracellular matrix, and cytokine networks in the treatment of osteoarthritis (Henry and O'Neill, 2025). However, there are bidirectional metabolic regulatory pathways between immune cells and stromal cells in OA (Nanus et al., 2021), This is consistent with our view that inflammatory factors can induce enhanced glycolysis and inhibited mitochondrial function in chondrocytes, while succinic acid, itaconic acid, and lactic acid secreted by chondrocytes and synovial cells, in turn, regulate the polarization of macrophages and the differentiation of T cells (Henry and O'Neill, 2025; Nanus et al., 2021). The current conceptual framework, which focuses solely on immunemetabolism, downplays to some extent the role of metabolic dysregulation in driving the metabolic reprogramming of immune cells, which hinders our understanding of the treatment of osteoarthritis as a whole.

Immune and metabolic reprogramming offers a new perspective on elucidating the mechanisms underlying OA treatment, but there remain significant barriers to clinical translation for targeted OA therapies, which severely limit the implementation of such treatments. The complex anatomical structure of joints presents multiple obstacles to targeted OA therapy: macromolecular drugs are restricted by the synovial barrier, and the avascular cartilage matrix hinders deep penetration. Furthermore, drugs must cross the cell membrane to precisely target mitochondria within cells (Conaghan et al., 2019; Zhang et al., 2026). Conventional intra-articular injections struggle to maintain effective local drug concentrations due to rapid clearance and low permeability; therefore, the development of targeted smart delivery systems has become an urgent priority. Current therapeutic strategies achieve targeted delivery by conjugating drugs with lipophilic cations or mitochondrial permeabilizing agents (Cao et al., 2021). However, achieving both efficient penetration of cartilage and precise delivery to mitochondria remains an unresolved technical challenge, one that depends on interdisciplinary breakthroughs in the fields of materials science and bioengineering.

Clinically, the etiology of OA varies significantly among patients—including mechanical injury, metabolic abnormalities, aging, and disease stage—and is associated with distinct molecular phenotypes. Consequently, therapeutic approaches targeting a single pathway may be effective only in specific patient populations. For example, promoting mitosis may improve age-related OA, but it has little effect in the treatment of traumatic OA (Zhang et al., 2026). Given this, it is necessary to classify patients based on biomarkers and implement personalized treatment. Furthermore, current research models of OA struggle to replicate the pathological characteristics of the disease in humans. Surgical models can only simulate acute mechanical osteoarthritis, whereas human osteoarthritis typically progresses gradually over the long term. Additionally, existing experimental studies often use young animals, thereby neglecting the impact of aging—a key factor—on mitochondria (Wang et al., 2023). These limitations undermine the predictive value of preclinical data regarding drug efficacy; therefore, there is a need for model systems that more closely mimic human physiology—such as humanized organoid models and models of induced aging—to enhance the value of clinical translation (Zhang et al., 2026).

In summary, current research on immunometabolism and metabolic reprogramming in osteoarthritis (OA) has largely focused on individual metabolic pathways or cell types, neglecting the interactive networks of multicellular metabolic dialogue within the joint microenvironment. At the translational level, progress has been hindered by species differences, model inaccuracies, and low efficiency of targeted delivery. Therefore, to overcome these bottlenecks, it is necessary to establish a multi-omics-based hierarchical strategy for immunometabolism and to develop precision delivery systems capable of simultaneously regulating both immune and metabolic nodes, thereby advancing research from conceptual exploration to clinical application.

### 5.4 Translational medicine

The clinical management of osteoarthritis is currently facing significant challenges. While anti-inflammatory analgesics and physical therapy can provide temporary symptom relief, they cannot stop the disease’s progression or reverse it. Intra-articular injections of corticosteroids or sodium hyaluronate may be required when the curative impact of oral medications is insufficient; however, these treatment methods increase the risk of side effects related to the gastrointestinal tract, kidneys, and heart (Talaie et al., 2022). Lastly, only high-risk perioperative joint replacement surgery can be accepted by patients in a critical sickness state (Talaie et al., 2022; Gill et al., 2003). Consequently, there is a significant treatment gap between conservative management and major surgery, which underscores the need to develop highly selective medications. For example, metformin is an oral medication that exerts its effects through systemic absorption. Researchers have found that combining it with celecoxib significantly reduces markers of cartilage degradation and inflammation in patients with knee osteoarthritis, thereby improving joint pain, stiffness, and physical function (Aiad et al., 2024). Another study found that MIV-711, a cathepsin K inhibitor, is capable of inhibiting bone resorption. Although a Phase IIa trial (244 patients with knee osteoarthritis, receiving 0/100/200 mg orally daily for 26 weeks) did not significantly alleviate pain, the 100 mg group significantly slowed the progression of articular cartilage thinning. Both the 100 mg and 200 mg doses inhibited pathological subchondral bone hyperplasia, and no increase in adverse events was observed. Subsequent analyses showed that the 100 mg dose significantly improved WOMAC pain scores in patients with unilateral knee pain; however, the developer subsequently completed only one Phase I safety trial and has not published the results or conducted further registration trials (Brandt et al., 2025). Based on this, while the oral regimen combining metformin and celecoxib is effective, systemic administration inevitably leads to off-target effects; the failure to develop an oral formulation of MIV-711 also underscores the need for localized, targeted delivery. Therefore, achieving precise delivery to the joint cavity may be a key strategy for enhancing therapeutic specificity and reducing systemic side effects.

Osteoarthritis is now a complicated molecular illness because of the development of biomarkers, which may allow for precise monitoring, categorization, and therapeutic procedures. As a result, the classification system for osteoarthritis may determine future trends. The key to achieving accurate osteoarthritis treatment is building predictive clinical models through the integration of clinical data and image analysis (Henry and O'Neill, 2025). In addition to being anticipated to independently prevent osteoarthritis joint destruction, it may also have synergistic effects with current rheumatoid arthritis biological agents (such as anti-tumor necrosis factor therapy) by focusing on key metabolites like lactic acid and succinic acid or by creating novel approaches to fully realize their therapeutic potential (Henry and O'Neill, 2025). Osteoarthritis is no longer thought of as a static state but rather as a complex molecular illness that can be accurately categorized, tracked, and treated thanks to advancements in biomarker research. Multidimensional osteoarthritis classification systems that integrate clinical data and imaging studies to create predictive clinical models will therefore likely be accepted by the field. To enable precise treatment for osteoarthritis, this integrated approach is essential.

In conclusion, the future of translational medicine for osteoarthritis depends on a thorough integration of fundamental research and clinical practice. Through a thorough examination of the metabolic network of osteoarthritis, precise targeting of important pathogenic nodes, and simultaneous optimization of drug molecular design and delivery systems, we have established the fundamental route to provide treatment with high specificity and minimal side effects. Thus, biomarkers enable the appropriate medication to be administered, completing the precision medicine closed loop. Therefore, the discovery of the intricate metabolic network underlying joint disorders has raised hopes for the creation of novel therapeutic approaches.

## 6 Conclusion

This review methodically demonstrates that the pathological remodeling that occurs in subchondral bone during osteoarthritis is a dynamic process driven by immunometabolic processes rather than a passive degenerative process. Local metabolic reprogramming occurs as a result of immune cell infiltration during this process. The steady-state balance of subchondral bone remodeling is upset by the combination of inflammatory stimuli and aberrant metabolic byproducts. This concept defines osteoarthritis as an immunometabolic illness, refuting the conventional notion that it is only “mechanical wear.” As a result, this departure from a mechanical perspective offers a fresh target for intervention as well as a theoretical basis for managing the illness.

The drawback of traditional treatment is that it can only lessen the symptoms of osteoarthritis; it cannot stop the illness from getting worse. In contrast, this review emphasizes that a promising therapeutic approach is to target the immunometabolic interaction network within subchondral bone. Targeting macrophages as the primary therapy target and transforming them from pro-inflammatory M1 type to anti-inflammatory repair-focused M2 type is anticipated to significantly interrupt the vicious cycle of inflammation and metabolic disorders among all related techniques. Consequently, this alteration has the potential to transform the harmful inflammatory microenvironment of subchondral bone into a condition that promotes healing. This suggests a shift in the way osteoarthritis is treated, moving from passive symptom management to active, mechanism-based, precise modulation of immunometabolism. This paves the way for the development of therapeutic strategies that can modify the course of the disease.

Currently, the complexity and variability of osteoarthritis cannot be adequately addressed by relying just on conventional single-subject research. Therefore, future research in immunology, metabonomics, and bone biology must transcend disciplinary boundaries through interdisciplinary collaboration and deep integration of knowledge and technology. As a result, interdisciplinary integration is now the key to unraveling OA’s pathogenic underpinnings and achieving ground-breaking treatment outcomes rather than a selected strategy for overcoming the condition.
